# WHAT ARE FAMILY CAREGIVERS NOT PREPARED FOR?

**DOI:** 10.1093/geroni/igae098.3929

**Published:** 2024-12-31

**Authors:** Roshni Singh, Saanvi Lamba, Richard Munoz, Pranjal Tyagi, Sandra Garcia-Davis, Stuti Dang, Diana Ruiz, VA Elizabeth Dole Center of Excellence

**Affiliations:** University of Miami, Miami, Florida, United States; Flint Hill School, Oakton, Virginia, United States; South Florida Veteran Affairs Foundation for Research and Education, Miami, Florida, United States; South Florida Veteran Affairs Foundation for Research and Education (SFVAFRE), Miami, Florida, United States; University of Miami, Miami, Florida, United States; Miami VA Healthcare System, Miami, Florida, United States; Miami Veterans Affairs Healthcare System, Miami, Florida, United States; VA, VA HSR, Texas, United States

## Abstract

Informal family caregivers of patients have a myriad of non-medical needs that are burdensome to caregivers and critical for disease management. Yet, caregivers are often not adequately prepared to address the needs of their Veterans. This study aimed to identify what caregivers feel unprepared for. We used round one data from the HERO CARE Survey. Here, we describe the preparedness of caregivers of Veterans, who responded to the following open-ended question: Out of all the tasks that you help the Veteran with, Is there anything specific you would like to be better prepared for? Qualitative responses to the open-ended question were coded and analyzed thematically. We received responses from 690 caregivers, 68.2 (SD: 12.2) years-old on average, 601 (87.1%) female, 70.7% non-Hispanic White, 62.0% spousal caregivers, 83.8% primary caregivers and 46.8% had provided care for >5 years. Twenty-one (3.0%) felt not prepared, 345 (50.0%) somewhat prepared, and 342 (49.6%) very prepared. Caregivers most important concerns reported were dealing with changes related to declining health (20.4%), equipment/home modifications (10.6%), dealing with memory loss (9.6%), advocating for services (8.8%), preparing for the future (7.8%), managing finances/legal matters (7.1%), mobility/falls/transfers (7.0%), respite/HHA (6.8%), handle emergencies (5.2%), and financial assistance (4.3%). Caregivers will be able to better meet the needs of patients if they are more prepared with the knowledge and skills to do their caregiving tasks. Developing ways to provide support, education, training, and resources will be pivotal in improving outcomes for veterans and their caregivers.

